# Convective Instabilities in Two Liquid Layers

**DOI:** 10.6028/jres.112.020

**Published:** 2007-10-01

**Authors:** G. B. McFadden, S. R. Coriell, K. F. Gurski, D. L. Cotrell

**Affiliations:** National Institute of Standards and Technology, Gaithersburg, MD 20899-8910; Department of Mathematics, George Washington Univesity, Washington, DC 20052; Lawrence Livermore National Laboratory, Livermore, CA 94550

**Keywords:** Benard convection, benzene-water system, fluid bilayers, hydrodynamic stability, Marangoni convection, Rayleigh-Taylor convection

## Abstract

We perform linear stability calculations for horizontal fluid bilayers, taking into account both buoyancy effects and thermocapillary effects in the presence of a vertical temperature gradient. To help understand the mechanisms driving the instability, we have performed both long-wavelength and short-wavelength analyses. The mechanism for the large wavelength instability is complicated, and the detailed form of the expansion is found to depend on the Crispation and Bond numbers. The system also allows a conventional Rayleigh-Taylor instability if heavier fluid overlies lighter fluid, and the long-wavelength analysis describes this case as well. In addition to the asymptotic analyses for large and small wavelengths, we have performed numerical calculations using materials parameters for a benzene-water system.

## 1. Introduction

The study of the stability of a fluid-fluid interface is important in a number of scientific and technological applications. In this paper we consider two immiscible fluid layers separated by a horizontal planar interface subject to a vertical temperature gradient. This problem has been well studied both theoretically and experimentally [1–6], and the effects of various driving forces on the stability of the system have been taken into account. Examples include the effects of buoyancy (natural or Rayleigh-Benard convection [7]), the effects of bulk density differences (Rayleigh-Taylor instabilities [8]), and the effects of surface tension gradients along the interface (Marangoni instabilities [1]).

One of the earliest papers on the two-layer problem was by Zeren and Reynolds [9], who carried out numerical calculations, a long wavelength analysis, and experiments on the benzene-water system. Given the limited computational resources at that time, Zeren and Reynolds obtained reasonable numerical results. With the increased computational power that is now available more accurate calculations may be performed, including the possibility of oscillatory (in time) modes that were ignored by Zeren and Reynolds. By using modern programs for symbol manipulation, the long wavelength analysis by Zeren and Reynolds can be extended to higher order as well.

In this paper we examine the linear stability of horizontal fluid bilayers, taking into account both buoyancy effects and thermocapillary effects in the presence of a vertical temperature gradient. The problem depends on a number of dimensionless parameters, including the ratio of material properties in each layer, the Rayleigh number (dimensionless temperature gradient), the Marangoni number (dimensionless force due to surface tension gradients), the Bond number (dimensionless buoyancy force at the interface), and the Crispation number (a measure of interface deformability). We find that in some situations the onset of instability can be due to a overstable mode that oscillates in time, with a finite critical wavenumber. In other circumstances the instability is stationary in time with a finite critical wavenumber. The stationary instabilities can extend to small wavenumbers, though the critical instability usually does not occur at zero wavenumber. To help understand the mechanisms driving the instability we have performed both long-wavelength and short-wavelength analyses. The mechanism for the large wavelength instability is complicated, and the detailed form of the expansion is found to depend on the Crispation and Bond numbers. The system allows a conventional Rayleigh-Taylor instability if heavier fluid overlies lighter fluid, and this case is included in the results as well. In addition to the asymptotic analyses for large and small wavenumbers, we have performed numerical calculations using materials parameters for a benzene-water system.

The paper is organized as follows. Governing equations are given in the next section. The numerical procedure is described briefly in Sec. 3. Linear stability results for various cases are presented next, including a comparison of the numerical results with large and small wavenumber expansions. A discussion is presented in Sec. 5, followed by conclusions in Sec. 6. An appendix contains a summary of the expansion results.

## 2. Equations

We consider a semi-infinite horizontal bilayer system, with vertical heating across the layers. The unperturbed upper layer (denoted by *α*) extends over the interval 0 < *z* < *H_α_*, and the unperturbed lower layer (denoted by *β*) extends over the interval − *H_β_* < *z* < 0. Without loss of generality we consider linear stability results for a two-dimensional system. The horizontal coordinate extends from − ∞ < *x* < ∞, and the velocity **u** has components in the *x* and *z* directions denoted by *u* and *w*, respectively.

### 2.1 Governing Equations in the Bulk

In each layer, we consider the Boussinesq equations

∇⋅u=0, (1)

$$\bar{\rho }u_{t}+\bar{\rho }(u\cdot \nabla )u+\nabla p=\mu \nabla^{2}u-\rho g\hat{z},$$ (2)

$$T_{t}+(u\cdot \nabla )T=\kappa \nabla^{2}T.$$ (3)

Here *p* is the pressure, *µ* is the dynamic viscosity, *T* is the temperature, *κ* is the thermal diffusivity, *g* is the gravitational acceleration, *t* is the time, and $\hat{z}$ is the unit vector in the *z*-direction (anti-parallel to gravity). We assume *µ* and *κ* are uniform in each layer, and also assume the density *ρ* is uniform in all terms except the gravitational term, where the density is given by

$$\rho =\bar{\rho }(1-\eta [T-T_{R}]),$$ (4)

in each layer. Here $\bar{\rho }$ is the density in each layer at the reference temperature *T_R_*, and the thermal expansion coefficient *η* is assumed to be uniform in each layer.

### 2.2 Boundary Conditions

The upper boundary at *z* = *H_α_* and the lower boundary at *z* = − *H_β_* are assumed to be isothermal with no-slip boundary conditions. The temperature is continuous across the interface,

〚T〛=0, (5)

where $〚T〛=T^{\alpha }-T^{\beta }$ denotes the temperature jump across the interface. The tangential velocity is assumed to satisfy the no-slip condition

$$〚u\cdot \hat{t}〛=0,$$ (6)

where $\hat{t}$ is any unit vector tangent to the interface. The stress boundary condition is

$$〚\bar{\rho }u(u\cdot \hat{n}-\upsilon_{n}〛=〚T\cdot \hat{n}〛-\gamma K\hat{n}+\nabla s\gamma ,$$ (7)

where $\hat{n}$ is a unit normal vector to the interface, $T_{jk}=-p\delta_{jk}+\mu (\partial u_{j}/\partial x_{k}+\partial u_{k}/\partial x_{j})$ is the stress tensor, γ is the surface tension, K is the curvature, *υ_n_* is the normal velocity of the interface, and ∇*s* is the surface gradient. Here our sign convention is that the curvature K is defined to be positive for a spherical inclusion of *β* phase. For example, in two dimensions with an interface *z* = *h*(*x, t*), the curvature is $K=-h_{xx}/{\left[1+h_{x}^{2}\right]}^{3/2}$, the interface velocity is $\upsilon_{n}=h_{t}/{\left[1+h_{x}^{2}\right]}^{1/2}$, and the surface gradient of the temperature-dependent surface energy *γ* = *γ*(*T*) is given by

$$\nabla s\gamma =\gamma_{T}\frac{\left(T_{x}+h_{x}T_{z}\right)}{\sqrt{1+h_{x}^{2}}}\hat{t},$$ (8)

where *γ_T_* = *dγ*/*dT* and $\hat{t}$ is the unit tangent vector to the interface in the direction of increasing *x*. Here *h_xx_* indicates the second derivative of *h*, etc.

The interface is a material surface, so that we have

$$u^{\alpha }\cdot \hat{n}=\upsilon_{n},$$ (9)

$$u^{\beta }\cdot \hat{n}=\upsilon_{n}.$$ (10)

Under these conditions the left hand side of Eq. (7) vanishes. The continuity of heat flux gives

$$〚k\frac{\partial T}{\partial n}〛=0,$$ (11)

where *k* is the thermal conductivity and $\partial T/\partial n=\left(T_{z}-h_{x}T_{x}\right)/\sqrt{1+h_{x}^{2}}$ is the normal derivative of the temperature field in each layer.

### 2.3 Base State

We linearize about a quiescent base state (also indicated by bars). The thermal field is

$${\bar{T}}^{\alpha }(z)=T_{E}+G_{\alpha }z,$$ (12)

in the *α* layer, and

$${\bar{T}}^{\beta }(z)=T_{E}+G_{\beta }z,$$ (13)

in the *β* layer, where *T_E_* is the unperturbed interface temperature. The temperature gradients in the base state satisfy

$$0=k_{\alpha }G_{\alpha }-k_{\beta }G_{\beta }.$$ (14)

The pressure field in the base state is hydrostatic, with

$$\frac{d{\bar{p}}^{\alpha }}{dz}=-{\bar{\rho }}_{\alpha }g,\;\frac{d{\bar{p}}^{\beta }}{dz}=-{\bar{\rho }}_{\beta }g.$$ (15)

### 2.4 Dimensionless Parameters and Governing Equations

Following the treatment by Zeren and Reynolds [9], we make the equations dimensionless based on a length scale given by the total depth *d* = *H_α_* + *H_β_*, a time scale based on the thermal time *d*^2^/*κ_β_*, a velocity scale *κ_β_*/*d*, a temperature scale *G_β_ d*, and a pressure scale $\nu_{\beta }\kappa_{\beta }{\bar{\rho }}_{\beta }/d^{2}.$ These scales introduce the dimensionless parameters

$$\nu^{*}=\frac{\nu_{\alpha }}{\nu_{\beta }},\;\rho^{*}=\frac{{\bar{\rho }}_{\alpha }}{{\bar{\rho }}_{\beta }},\;\eta^{*}=\frac{\eta_{\alpha }}{\eta_{\beta }}$$ (16)

$$\kappa^{*}=\frac{\kappa_{\alpha }}{\kappa_{\beta }},\;k^{*}=\frac{k_{\alpha }}{k_{\beta }},\;G^{*}=\frac{G_{\alpha }}{G_{\beta }},\;\mu^{*}=\frac{\mu_{\alpha }}{\mu_{\beta }},$$ (17)

$$\begin{array}{l} \;\mathrm{Pr}=\frac{\nu_{\beta }}{\kappa_{\beta }},\;\text{Ra}=\frac{g\eta_{\beta }G_{\beta }d^{4}}{\nu_{\beta }\kappa_{\beta }},\;\text{Cr}=\frac{\mu_{\beta }\kappa_{\beta }}{d\gamma },\\ \text{Bo}=\frac{g\rho_{\beta }d^{2}}{\gamma },\;\text{Ma}=\frac{-\gamma_{T}G_{\beta }d^{2}}{\mu_{\beta }\kappa_{\beta }}, \end{array}$$ (18)

and the geometrical parameter $\ell =-H_{\beta }/H_{\alpha }$. Here $\nu_{\alpha }=\mu_{\alpha }/{\bar{\rho }}_{\alpha }$ and $\nu_{\beta }=\mu_{\beta }/{\bar{\rho }}_{\beta }$ are the kinematic viscosities in each layer, Pr is the Prandtl number, Ra is the Rayleigh number, Cr is the Crispation number, Bo is the Bond number, and Ma is the Marangoni number. The minus sign in the definition of Ma is conventional, since for most materials, as for the benzene-water system, we have *γ_T_* < 0.

We assume a horizontal wavenumber *a* and a temporal growth rate *σ* = *σ_r_* + *iσ_i_*. The perturbed quantities (indicated by tildes) then satisfy

$$ia{\tilde{u}}^{\alpha }+{\tilde{\omega }}_{z}^{\alpha }=0,$$ (19)

$${\mathrm{Pr}}^{-1}\sigma {\tilde{u}}^{\alpha }+ia{\tilde{p}}^{\alpha }/\rho^{*}=\nu^{*}\left({\tilde{u}}_{zz}^{a}-a^{2}{\tilde{u}}^{\sigma }\right),$$ (20)

$${\mathrm{Pr}}^{-1}\sigma {\tilde{\omega }}^{\alpha }+{\tilde{p}}_{z}^{\alpha }/\rho^{*}=\nu^{*}\left({\tilde{\omega }}_{zz}^{\alpha }-a^{2}{\tilde{\omega }}^{\alpha }\right)+\eta^{*}\text{Ra}\;{\tilde{T}}^{\alpha },$$ (21)

$$\sigma {\tilde{T}}^{\alpha }+G^{*}{\tilde{\omega }}^{\alpha }=\kappa^{*}\left({\tilde{T}}_{zz}^{\alpha }-a^{2}{\tilde{T}}^{\alpha }\right),$$ (22)

for *z* > 0, and

$$ia{\tilde{u}}^{\beta }+{\tilde{\omega }}_{z}^{\beta }=0,$$ (23)

$${\mathrm{Pr}}^{-1}\sigma {\tilde{u}}^{\beta }+ia{\tilde{p}}^{\beta }={\tilde{u}}_{{}^{zz}}^{\beta }-a^{2}{\tilde{u}}^{\beta },$$ (24)

$${\mathrm{Pr}}^{-1}\sigma {\tilde{\omega }}^{\beta }+{\tilde{p}}_{z}^{\beta }={\tilde{\omega }}_{zz}^{\beta }-a^{2}{\tilde{\omega }}^{\beta }+\text{Ra}\;{\tilde{T}}^{\beta },$$ (25)

$$\sigma {\tilde{T}}^{\beta }+{\tilde{\omega }}^{\beta }={\tilde{T}}_{zz}^{\beta }-a^{2}{\tilde{T}}^{\beta },$$ (26)

for *z* < 0.

The boundary conditions at *z* = 0 are

$${\tilde{T}}^{\alpha }+G^{*}\tilde{h}={\tilde{T}}^{\beta }+\tilde{h},$$ (27)

$${\tilde{u}}^{\alpha }-{\tilde{u}}^{\beta }=0,$$ (28)

$$\left({\tilde{p}}^{\alpha }-{\tilde{p}}^{\beta }\right)-{\text{BoCr}}^{-1}\left(\rho^{*}-1\right)\tilde{h}+a^{2}{\text{Cr}}^{-1}\tilde{h}=2\left(\mu^{*}{\tilde{\omega }}_{z}^{\alpha }-{\tilde{\omega }}_{z}^{\beta }\right),$$ (29)

$$\left(\mu^{*}{\tilde{\mu }}_{z}^{\alpha }-{\tilde{\mu }}_{z}^{\beta }\right)+ia\left(\mu^{*}{\tilde{\omega }}^{\alpha }-{\tilde{\omega }}^{\beta }\right)-ia\text{Ma}\left({\tilde{T}}^{\alpha }+G^{*}\tilde{h}\right)=0,$$ (30)

$${\tilde{\omega }}^{\alpha }=\sigma \tilde{h},$$ (31)

$${\tilde{\omega }}^{\beta }=\sigma \tilde{h},$$ (32)

$$k^{*}{\tilde{T}}_{z}^{\alpha }={\tilde{T}}_{z}^{\beta }.$$ (33)

Critical conditions are often determined experimentally by varying the temperature gradient across the system. The temperature gradient *G_β_* appears in the dimensionless parameters Ma and Ra. Zeren and Reynolds [9] introduce the parameter

$$\Gamma =\frac{\text{Ra}}{\text{Ma}}=\frac{-\rho g\eta_{\beta }d^{2}}{\gamma_{T}},$$ (34)

which is independent of the temperature gradient, and perform calculations by varying Ma for a fixed value of Γ.

## 3. Numerical Implementation

We solve the eigenvalue problem that governs the linear stability of the system by using two complementary procedures. In the first approach, the equations are discretized using pseudo-spectral Chebyshev collocation, and the resulting generalized matrix eigenvalue problem is solved using the package RGG from the EISPACK software library [10]. For a discretization with *N* degrees of freedom, this routine produces approximations to the first *N* eigenvalues of the system. The second approach is to use the two-point boundary value solver BVSUP [11], coupled with the root finder SNSQ [12], both from the SLATEC library [13], to implement a method described by Keller [14] to solve the eigenvalue problem. The BVSUP procedure provides a very accurate solution for a given eigenmode provided a good enough initial estimate is available for the root-finding procedure. The pseudospectral method is efficient for small values of *N*, and is well-suited for searching parameter space to detect real and complex eigenvalues. Rather than performing fine grid calculations with the pseudospectral procedure, however, the coarse grid results from the pseudospectral method are often used as initial guesses for the BVSUP code. Continuation from previous solutions also is used once an eigenmode has been identified.

The BVSUP software works in a single domain, so we have mapped the two layers to a common domain by setting

$$\bar{z}=\{\begin{array}{cc} z & \text{for}\;0<z<H_{\alpha },\\ -H_{\alpha }z/H_{\beta } & \;\text{for}\;-H_{\beta }<z<0, \end{array}$$ (35)

so that $0<\bar{z}<H_{\alpha }$ in each layer. We then have

$$\frac{d}{dz}=\{\begin{array}{cc} d/d\bar{z} & \text{for}\;0<z<H_{\alpha },\\ (1/\ell )d/d\bar{z} & \;\text{for}\;-H_{\beta }<z<0, \end{array}$$ (36)

where $\ell =-H_{\beta }/H_{\alpha }$. To simplify the treatment of the problem, we also introduce an auxiliary ordinary differential equation in $\bar{z}$ for the interface $\tilde{h}$, by setting

$$\frac{d\tilde{h}}{d\bar{z}}=0,$$ (37)

which allows us to avoid eliminating $\tilde{h}$ as an unknown from the interface boundary conditions.

## 4. Results

In this section we present numerical results for the linear stability of the bilayer system. We provide a comparison with previous results of Zeren and Reynolds [9], who consider benzene overlying water. We use the thermophysical parameters given in their Table II for a temperature of 16 °C, and also consider their case $d_{b}^{*}=H_{\beta }/\left(H_{\alpha }+H_{\beta }\right)=0.4$. Zeren and Reynolds consider both the case of heating from above (positive Ma), where the main driving force is the Marangoni effect and buoyancy effects are expected to be stabilizing, and the case of heating from below (negative Ma), where both buoyancy effects and Marangoni effects can produce instabilities.

If the system is heated from above, Zeren and Reynolds compute a positive critical Marangoni number of 1486, with a critical wavenumber of *a* = 2.6; they consider only stationary modes with *σ_i_* = 0. For *a* = 2.6 we find Ma = 1468.4635, in fair agreement with their results. We find a critical wavenumber to three digits of *a* = 2.66, with Ma = 1466.8951. Ferm and Wollkind [15] also obtained similar agreement in a comparison with Zeren and Reynolds, although they chose different thermophysical properties for the benzene-water system.

**Fig. 1:**
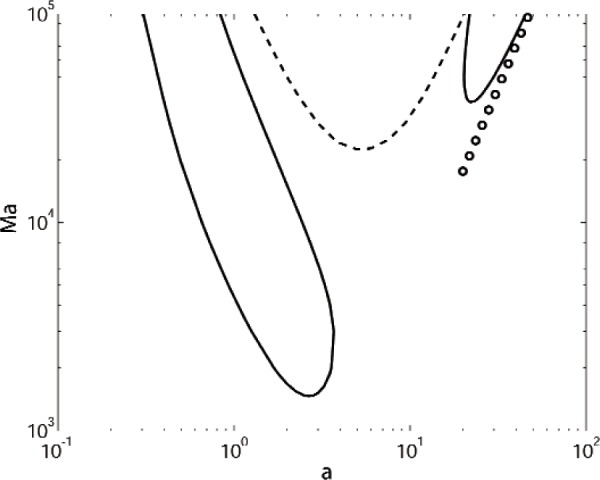
Marginal stability curves of Marangoni number Ma versus wavenumber *a* for a system of benzene over water with heating from above. Solid curves represent stationary modes with *σ_i_* = 0, and the dashed curve is an oscillatory mode with *σ_i_* ≠ 0. The circles denote the results of the large wavenumber expansion.

Our computed neutral stability curves for this case are shown in Fig. 1. As discussed by Zeren and Reynolds, the critical mode is mainly driven by Marangoni convection. We show the two stationary modes that are given in the second figure of Zeren and Reynolds; the convection pattern in these modes has a single cell in each layer, with negligible surface deformation. The convection is more concentrated near the interface for the higher wavenumber mode. We also find an oscillatory mode (*σ_i_* ≠ 0) at intermediate wavenumbers and Marangoni numbers. The oscillatory mode has a minimum near *a* = 5.5, with Ma = 22379.593 and *σ_i_* = ± 165.1. The surface deflection for this mode is negligible. The critical branch of the stationary mode does not extend to very small wavenumbers, but instead shows a large-Ma asymptote as the wavenumber tends to the value *a* = 0.12.

**Fig. 2:**
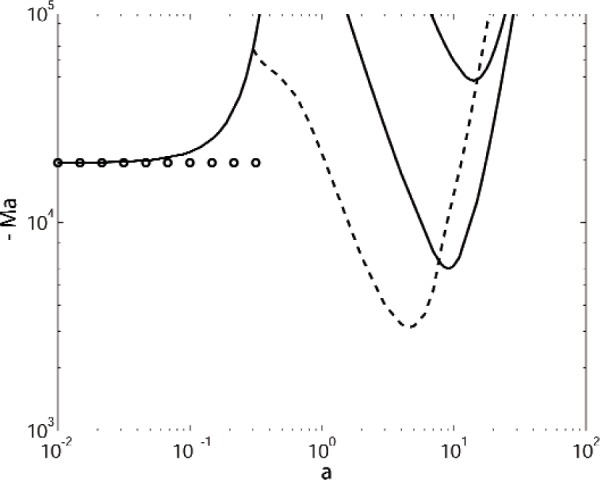
Marginal stability curves for a system of benzene over water with heating from below. Solid curves represent stationary modes with *σ_i_* = 0, and the dashed curve is an oscillatory mode with *σ_i_* ≠ 0. The circles denote the limiting value of the small wavenumber expansion.

If the system is heated from below, Zeren and Reynolds compute a negative critical Marangoni number of Ma = − 6068, with a critical wavenumber of *a* = 9. For *a* = 9.0 we find Ma = − 6014.4082. We show our computed marginal stability curves for this case in Fig. 2. We find that the critical mode is actually oscillatory, with Ma = − 3146.7277 for *a* = 4.6 and *σ_i_* = ± 64.93. The surface deflection for this mode is negligible. This mode exhibits a single convective cell in the upper layer, and two vertically-stacked cells in the lower layer of comparable strength. This mode merges with another stationary branch that has a long-wavelength asymptote; *σ_i_* tends to zero on the oscillatory branch as the modes merge. On the long-wavelength branch, for *a* = 0.001 we find Ma ≈ − 19318.282. Analytic results for long-wavelength modes are described in the appendix; for this case the asymptotic result is Ma = − 19318.06, in excellent agreement with the numerical results.

There is a single convection cell in each layer for the long wavelength mode, with a significant interface deflection. Examination of the eigenmode shows that there is down-flow in the layer beneath the elevated portion of the perturbed interface. As discussed by Scriven and Sternling [16] for the single layer case, Marangoni modes and buoyant modes can be distinguished by the direction of the vertical flow in the layer beneath an interface elevation: there is downflow beneath elevations for Marangoni flow, and upflow beneath elevations for buoyancy-driven flow. This is consistent with the Zeren and Reynold’s interpretation of the long-wavelength mode as driven by Marangoni effects. The other two stationary modes in Fig. 2 with minima near *a* = 10 have negligible surface deflection. For both modes the lower layer is nearly isothermal with a weak unicellular flow, and the upper layer exhibits two vertically-stacked convective cells for the mode with Ma ≈ − 6000, and three vertically-stacked convective cells for the mode with Ma ≈ − 50,000. There is in fact an entire family of additional stationary buoyant modes, not shown in Fig. 2, with larger values of | Ma |, each containing multiple vertically-stacked cells in the two layers.

## 5. Discussion

**Fig. 3:**
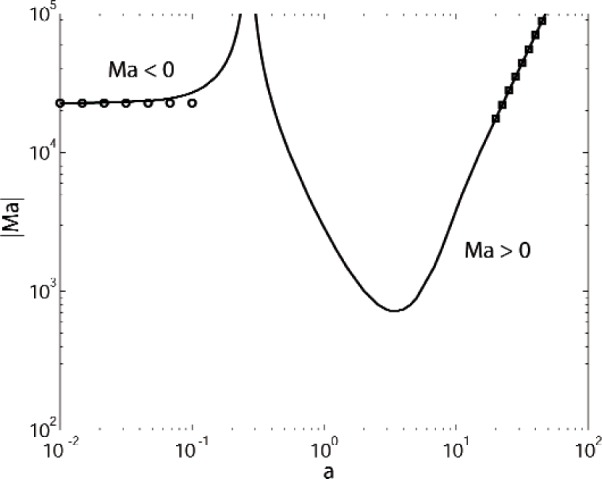
Marginal stability curves for a system of benzene over water with buoyancy suppressed (Γ = 0). The circles denote the limiting value of the small wavenumber expansion, and the squares denote the results of the large wavenumber expansion.

For the case of heating from below, as shown in Fig. 2 there is a small wavenumber instability that asymptotically approaches a finite Marangoni number as the wavenumber *a* tends to zero. To examine this behavior in more detail, we have performed additional calculations with Γ = 0, which eliminates the effects of buoyancy. Results are shown in Fig. 3 for both heating from below (Ma < 0) and for heating from above (Ma > 0). Suppressing buoyancy eliminates the oscillatory modes that prevail at intermediate wavenumbers for Γ = 0.142. For heating from below with our parameter values the stationary mode has a small-wavenumber limiting behavior that is given approximately by the expression

$$-\text{Ma}=\frac{2.2691\times 10^{4}(1+7.6026a^{2})}{1-8.8850a^{2}}+O(a^{4}),$$ (38)

(see Eq. (51) in the appendix). Note that in this approximation there is a pole at *a* = 0.335. This is in approximate agreement with the vertical asymptote that is obtained numerically for *a* = 0.268 for heating from either above or below. In fact, plotting instead 1/Ma versus *a* produces a single smooth curve crossing the *y*-axis with 1/Ma = 0 at a wavenumber *a* = 0.268.

**Fig. 4:**
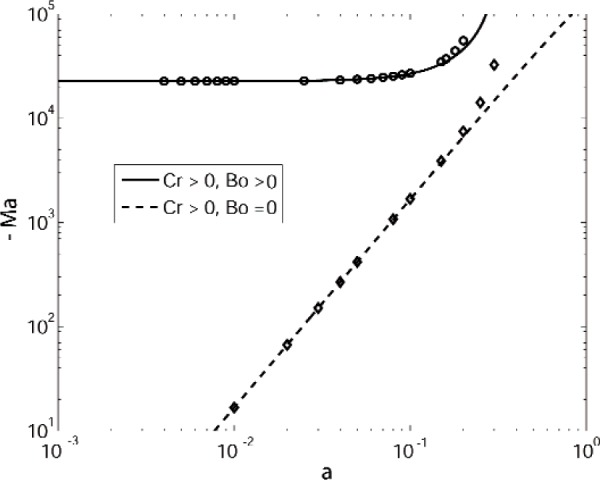
Marginal stability curves for a system of benzene over water with buoyancy suppressed (Γ = 0). The circles indicate computational results obtained for the parameter values given in Table I, and the solid curve denotes the results of the corresponding small wavenumber expansion. The diamonds indicate computational results obtained with Bo = 0, and the dashed curve denotes the results of the corresponding small wavenumber expansion.

To help further understand the low wavenumber instability that is observed numerically, we have also performed numerical computations and asymptotic expansions that illustrate the effects of the Bond number Bo and the Crispation number Cr for this mode. In Fig. 4 the upper curve corresponds to the data given in Table I. This curve asymptotically approaches a small wavenumber limit for Ma = − 2.27 × 10^4^. The lower curve corresponds to setting Bo = 0. The symbols on the curves correspond to numerical results, and the curves themselves correspond to analytical results from the small wavenumber approximation given in the appendix. The small wavenumber results depend strongly on both Bo and Cr. For the parameter values given in Table I, we have Cr = 2.1724 × 10^−6^ and Bo = 1.1905, giving

$$-\text{Ma}\approx \frac{c_{1}[1-\rho^{*}]\text{Bo}}{c_{2}\text{Cr}}=2.2691\times 10^{4},$$ (39)

and for Cr = 2.1724 × 10^−6^ and Bo = 0,

$$-\text{Ma}\approx \frac{c_{1}}{c_{2}\text{Cr}}=1.67190\times 10^{5}a^{2}.$$ (40)

**Fig. 5:**
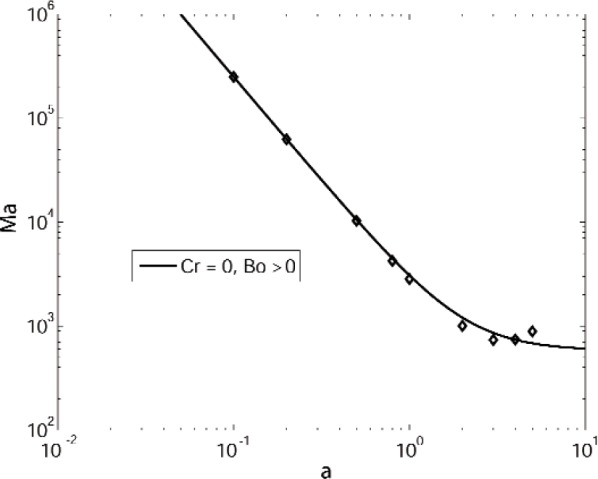
Marginal stability curves for a system of benzene over water with buoyancy suppressed (Γ = 0). The diamonds indicate computational results obtained with Cr = 0, and the solid curve denotes the results of the corresponding small wavenumber expansion.

For Cr = 0 and Bo = 1.1905, the resulting Marangoni number is positive (corresponding to heating from above). with

$$\text{Ma}\approx \frac{c_{1}}{c_{4}}a^{-2}+\frac{c_{6}}{c_{4}}=\frac{2.4909\times 10^{3}}{a^{2}}+583.1869,$$ (41)

and numerical results for this case are shown in Fig. 5. Here the coefficients *c*_1_, *c*_2_, *c*_4_, and *c*_6_ in these expressions generally depend on the layer geometry and the remaining material constants and are given in the appendix; here we have evaluated these expressions for the values given in Table I.

### 5.1 Large Wavenumbers

**Fig. 6:**
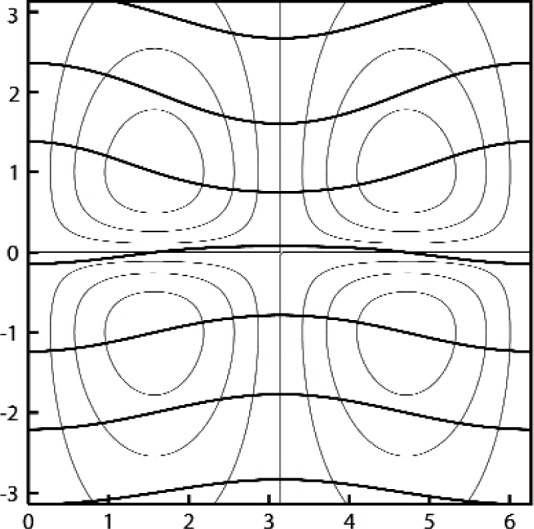
Streamfunction contours (light lines) and temperature contours near the interface for the large wavenumber Marangoni instability with *a* = 1, k* = 1.0. and κ* = 0.5. The magnitude of the perturbation is exaggerated to emphasize the deformation of the temperature contours.

The temperature and flow field for the Marangoni instability can be studied in the large wavenumber limit that is summarized in the appendix. We ignore buoyancy by setting Γ = 0, and also ignore interface deformation by setting Cr = 0, which the numerical results indicate are good approximations in this case. In the large wavenumber limit the flow is confined to the vicinity of the interface and the effect of the upper and lower boundaries at *z* = *H_α_* and *z* = − *H_β_* is negligible. To help visualize the flow we introduce a two-dimensional streamfunction *ψ* with *w* =*ψ_x_* and *u* = −*ψ_z_*. Contours of the temperature and streamfunction near the interface are shown in Fig. 6. Here we have exaggerated the size of the perturbation to emphasize the distortion of the isotherms near the interface. To make the plots easier to interpret we have assumed equal thermal conductivities (k* = 1), which equalizes the unperturbed temperature gradients in the two layers and facilitates comparison of the perturbed temperature fields in each layer. There is a temperature gradient along the interface (at *z* = 0), and the streamlines of the flow are along the interface. Over the single period of the flow shown in Fig. 6, there are four convective cells near the interface that alternately compress and expand the isotherms near the interface. For our parameters with κ* < 1, the Marangoni instability occurs for heating from above (Ma > 0) in the large wavenumber limit. If κ* > 1 the instabilities occur for heating from below (Ma < 0) instead.

### 5.2 Rayleigh-Taylor Instability

The two-layer system can exhibit the classical Rayleigh-Taylor instability if heavier fluid overlies lighter fluid. In the simplest case the Rayleigh-Taylor instability can be understood by a simple potential energy argument that balances the increased surface energy of a deformed interface *y* = *h*(*x*) against the change in the gravitational potential energy of the displaced fluid,

$$g({\bar{\rho }}_{\alpha }-{\bar{\rho }}_{\beta })h=-\gamma h_{xx}.$$ (42)

In terms of our dimensionless variables, this takes the form

$$-\text{Bo}=\frac{a^{2}}{\left(1-\rho^{*}\right)},$$ (43)

which can be seen as a factor in the normal stress balance boundary condition (29).

**Fig. 7:**
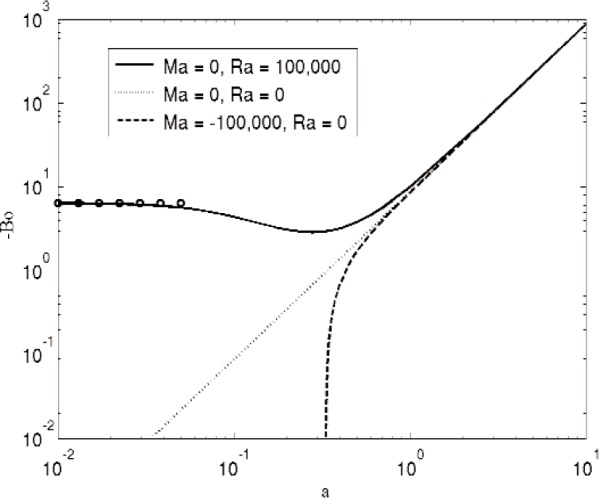
Marginal stability curves for a water-benzene system that is unstably stratified with respect to gravity. The dotted curve (Ma = Ra = 0) corresponds to the classical Rayleigh-Taylor instability in the absence of buoyancy, given by − Bo = *a*^2^/(1 − ρ*). The solid curve represents numerical results that include the effects of buoyancy, with Ra = 1.0 × 10^5^ and Ma = 0, and the circles are the small wavenumber asymptotic results for this value of Ra. The dashed curve represents numerical results that include the Marangoni effect, with Ra = 0 and Ma = − 1.0 × 10^5^.

In the situation we have studied above, we have lighter benzene overlying heavier water, so the Rayleigh-Taylor instability does not occur. To study this instability for our system with a minimal change in notation, we temporarily choose to change the direction of gravity while keeping benzene and water in the original orientation, so that the water and benzene are unstably stratified with respect to gravity. We take *G_β_* < 0, so that buoyancy has a stabilizing effect on the system; the resulting sign conventions produce Ma < 0, Ra > 0, Γ < 0, and Bo < 0. In Fig. 7 we show the corresponding numerical results for the Rayleigh-Taylor instability. The dotted curve in Fig. 7 shows the curve −Bo = *a*^2^/(1 − ρ*) that holds for Ra = Ma = 0. The solid curve shows numerical results for Ra = 1.0 × 10^5^ and Ma = 0. The stabilizing effect of buoyancy is evident at small wavenumbers, where the system is then stable if | Bo | is sufficiently small. The marginal stability curve asymptotes at small wavenumbers to the value

$$\text{Bo}=\frac{-c_{5}\text{CrRa}}{c_{1}(1-\rho^{*})}\approx -6.45$$ (44)

in our case, which follows from Eq. (45) in the appendix. The dashed curve shows numerical results for Ra = 0 and Ma = − 1.0 × 10^5^. The destabilizing Marangoni effect in this configuration is evident at small wavenumbers. and there is a cut-off wavenumber at *a* = 0.333 where Bo = 0; at lower wavenumbers this mode merges into the Marangoni instability with positive Bond numbers. This value of the cut-off wavenumber is too large to be quantitatively approximated by the small wavenumber expansion given by Eq. (51) in the appendix. For the benzene-water system with Ra/Ma = − 0.142, numerical results are qualitatively similar to those for the case with Ra = 0.

Our small wavenumber expansion of the instability, as summarized in the appendix, corrects and extends a similar analysis given by Zeren and Reynolds [9], who note that at leading order in *a* only the interface position and temperature field are perturbed, with no leading order velocity profiles. However, the asymptotic values for the critical Marangoni number do depend on the viscosity of the fluid, so flow does have an effect on the stability of the system, even at small wavenumbers. The small wavenumber analysis proceeds by an expansion in powers of *a*^2^, and the critical Marangoni number is determined at a later point in the expansion where the *O*(*a*^2^) flow perturbation is significant. We also note that the expansion procedure is based on a limit of small but non-zero wavenumbers. In the special one-dimensional case *a* = 0 the perturbed interface is flat and mass conservation restricts its location to *z* = 0. In a sense this represents a discontinuity in the problem in the limit *a* → 0, since in the expansion procedure a shift in the interface location is obtained at leading order. This is somewhat analogous to the dependence of a perturbed interface of the form *z* = *H*_0_ cos *a x*, which conserves mass for *a* ≠ 0 (i.e., the average interface position is zero), but represents a shifted flat interface at *z* = *H*_0_ for *a* = 0. In general, for real experiments the smallest allowable value of *a* will be limited by the finite lateral extent of the system, but whether or not a perturbation with *a* = 0 is physically relevant depends on the specific problem under consideration.

## 6. Conclusions

We have performed linear stability calculations for horizontal fluid bilayers, taking into account both buoyancy effects and thermocapillary effects. We consider the case of a lighter layer overlying a heavier layer, so that the base state is stably stratified in this sense. We find that the system can be linearly unstable to either heating from above (Ma > 0) or below (Ma < 0).

The mechanism for the large wavelength (small *a*) instability is complicated (see Figs. 4 and 5). The detailed form of the expansion, viz, the exponent *n* in the leading order expansion Ma ∼ *a*^n^ depends on the Crispation and Bond numbers.

## Figures and Tables

**Table I:** Dimensionless parameters for the benzene (*α* layer) and water (*β* layer) system at *T* = 16 °C, from Ref. [9]

ratio of densities	$\rho^{*}={\bar{\rho }}_{\alpha }/{\bar{\rho }}_{\beta }$	0.886
ratio of dynamic viscosities	µ^*^ = *µ_α_*/*µ_β_*	0.605
ratio of thermal conductivities	k^*^ = *k_α_*/*k_β_*	0.274
ratio of thermal diffusivities	κ^*^ = *κ_α_*/*κ_β_*	0.730
ratio of thermal expansion coefficients	η^*^ = *η_α_*/*η_β_*	7.06
Bond number over Crispation number	Bo/Cr	5.48(10^5^)
Inverse Bond number	Bo^−1^	0.840
Prandtl number	Pr	8
Rayleigh number over Marangoni number	Γ	0.142
dimensionless thickness ratio	*H_α_*/*H_β_*	1.5

## 7. Appendix

Here we consider the limits of large and small wavenumbers. We introduce the dimensionless layer widths ${\bar{H}}_{\alpha }=H_{\alpha }/d=1/(1-\ell )$ and ${\bar{H}}_{\beta }=H_{\beta }/d=-\ell /(1-\ell )$, where *d* = *H_α_* + *H_β_* and $\ell =-H_{\beta }/H_{\alpha }=-2/3$ in our calculations.

### 7.1 Small Wavenumbers

By performing long-wavelength asymptotics for Bo ≠ 0 and Cr ≠ 0 we find the leading order result

$$\text{Ma}=\frac{c_{1}\text{Bo}(\rho^{*}-1)}{\text{Cr}(c_{2}+c_{5}\Gamma )},$$ (45)

where

$$c_{1}=80{\bar{H}}_{\alpha }{\bar{H}}_{\beta }[{\bar{H}}_{\alpha }+k^{*}{\bar{H}}_{\beta }][{\bar{H}}_{\alpha }+\mu^{*}{\bar{H}}_{\beta }],$$ (46)

$$c_{2}=120[{\bar{H}}_{\alpha }^{2}-\mu^{*}{\bar{H}}_{\beta }^{2}]$$ (47)

$$\begin{array}{c} c_{5}=\frac{2(1-k^{*})}{k^{*}}\{11{\bar{H}}_{\alpha }^{2}{\bar{H}}_{\beta }^{2}\left[\eta^{*}\mu^{*}\rho^{*}{\bar{H}}_{\alpha }-k^{*}{\bar{H}}_{\beta }\right]\\ +3\left[\eta^{*}\rho^{*}{\bar{H}}_{\alpha }^{5}-k^{*}\mu^{*}{\bar{H}}_{\beta }^{5}\right]+14{\bar{H}}_{\alpha }{\bar{H}}_{\beta }\left[\eta^{*}\rho^{*}{\bar{H}}_{\alpha }^{3}-k^{*}\mu^{*}{\bar{H}}_{\beta }^{3}\right]\}. \end{array}$$ (48)

This expression is in good agreement with the numerical results for small wavenumbers as shown in Fig. 2. For Ra = 0, this expression agrees with that given by Zeren and Reynolds [9] up to a difference in sign; their expression also agrees with their numerical results for Ra = 0 if the sign of their expression is changed. For Ra ≠ 0 our expression gives results which differ in both magnitude and sign from that given by Zeren and Reynolds. We note that the leading order result in Eq. (45) is proportional to Bo and inversely proportional to Cr, so that the special cases Bo = 0 or Cr = 0 require additional attention; we consider these cases in more detail below for Ra = 0.

For the values given in Table I, we have

$$c_{1}=11.4717,\;c_{2}=31.5840,\;c_{5}=38.8300.$$ (49)

We note that in general *c*_l_ is positive, but *c*_2_ can change sign depending on the magnitude of *µ**, ${\bar{H}}_{\alpha }$, and ${\bar{H}}_{\beta }=1-{\bar{H}}_{\alpha }$. For a fixed layer geometry (given ${\bar{H}}_{\alpha }$), large and small values of *µ** thus produce a sign change in the asymptotic value of Ma for small *a* and Γ = 0; this corresponds to the dominant flow occurring primarily in either the upper or lower layer as the corresponding viscosity contrast is large. The expression (48) for *c*_5_ is more complicated and also can have either sign, depending on the parameter values.

#### 7.1.1 Case for Ra = 0

If we set Ra = 0 the linear eigenfunctions for the general problem can be written down explicitly, and the dispersion relation for *σ* = 0 can be evaluated symbolically in closed form, though the resulting expression is lengthy and difficult to interpret. However, the result can be expanded for small wavenumbers, and produces expressions that are not too unwieldy.

The general expression for *σ* = 0 and Ra = 0 can be found by expanding in the wavenumber *a* to give

$$\begin{array}{c} 0=a^{10}\{c_{1}[\rho^{*}-1]\text{Bo}-c_{2}\text{CrMa}\}\\ +a^{12}\{-\text{Ma}(c_{3}\text{Cr}+c_{4}[\rho^{*}-1]\text{Bo})-(c_{1}-c_{6}[\rho^{*}-1]\text{Bo})\}+O(a^{14}). \end{array}$$ (50)

The expression can be solved for Ma to give a rational expression of the form

$$\text{Ma}=\frac{c_{1}[\rho^{*}-1]\text{Bo}-(c_{1}-c_{6}[\rho^{*}-1]\text{Bo})a^{2}}{c_{2}\text{Cr}+(c_{3}\text{Cr}+c_{4}[\rho^{*}-1]\text{Bo})a^{2}}+O(a^{4}).$$ (51)

The constants *c*_l_ and *c*_2_ are as given above, and the additional constants *c*_3_, *c*_4_, and *c*_6_ are given by

$$c_{3}=4(9[{\bar{H}}_{\alpha }^{4}-\mu^{*}{\bar{H}}_{\beta }^{4}]+10{\bar{H}}_{\alpha }{\bar{H}}_{\beta }[{\bar{H}}_{\alpha }^{2}-\mu^{*}{\bar{H}}_{\beta }^{2}]+5{\bar{H}}_{\alpha }^{2}{\bar{H}}_{\beta }^{2}[1-\mu^{*}])$$ (52)

$$c_{4}=\frac{{\bar{H}}_{\alpha }^{3}{\bar{H}}_{\beta }^{3}}{\kappa^{*}}[{\bar{H}}_{\alpha }^{2}-\kappa^{*}{\bar{H}}_{\beta }^{2}]$$ (53)

$$\begin{array}{l} c_{6}=\frac{8}{3}{\bar{H}}_{\alpha }{\bar{H}}_{\beta }(9[{\bar{H}}_{\alpha }^{4}+k^{*}\mu^{*}{\bar{H}}_{\beta }^{4}]+21{\bar{H}}_{\alpha }^{2}{\bar{H}}_{\beta }^{2}[1+k^{*}\mu^{*}]\\ \;+19{\bar{H}}_{\alpha }{\bar{H}}_{\beta }[k^{*}{\bar{H}}_{\alpha }^{2}+\mu^{*}{\bar{H}}_{\beta }^{2}]+11{\bar{H}}_{\alpha }{\bar{H}}_{\beta }[k^{*}{\bar{H}}_{\beta }^{2}+\mu^{*}{\bar{H}}_{\alpha }^{2}]). \end{array}$$ (54)

We note that the dependence on the Bond number Bo enters through the quantity [*ρ** − 1]Bo − *a*^2^. For the values given in Table I, the additional constants are given by

$$c_{3}=7.0898,\;c_{4}=4.6055\times 10^{-3},\;c_{6}=2.68.59.$$ (55)

Note that if Cr = 0 in Eq. (51) the two-term approximation,

$$\text{Ma}=\frac{c_{1}}{c_{4}}a^{-2}+\frac{c_{6}}{c_{4}},$$ (56)

is independent of Bo. This is a result of the boundary condition (29); for Cr = 0, the interface is rigid and the Bond number has no effect on the system.

### 7.2 Large Wavenumbers

We next consider the limit of large wavenumbers for a stationary mode. For our system the numerical results suggest that buoyancy effects and interface deformation are unimportant in this limit, so we also consider the formal limit of small Crispation number, Cr → 0, along with Ra = 0. For Ra = 0, the governing equations for the velocity field are decoupled from the thermal field, which simplifies the analysis. For Cr → 0 the dimensionless form of the normal momentum balance,

$$\text{Cr}({\tilde{p}}^{\alpha }-{\tilde{p}}^{\beta })-\text{Bo}(\rho^{*}-1)\tilde{h}+a^{2}\tilde{h}=2\text{Cr}(\mu^{*}{\tilde{\omega }}_{z}^{\alpha }-{\tilde{\omega }}_{z}^{\beta }),$$ (57)

then reduces to

$$\text{Bo}(\rho^{*}-1)\tilde{h}-a^{2}\tilde{h}=0.$$ (58)

For Bo (*ρ** − 1) − *a*^2^ ≠ 0, we conclude that the interface deformation vanishes, $\tilde{h}=0$.

In the limit of large wavenumber the disturbances are concentrated near the interface and the effects of the outer boundaries are insignificant. The appropriate solution can then be computed by applying decay conditions in an unbounded domain as *z* → ± ∞. The vertical components of the velocity field are given by

$${\tilde{w}}^{\alpha }(z)=A_{\alpha }e^{-az}+B_{\alpha }aze^{-az},\;{\tilde{w}}^{\beta }(z)=A_{\beta }e^{az}+B_{\beta }aze^{az}.$$ (59)

The temperature fields are given by

$$\begin{array}{c} {\tilde{T}}^{\alpha }(z)=\frac{-G^{*}A_{\alpha }}{2a^{2}\kappa^{*}}[az]e^{-az}-\frac{G^{*}B_{\alpha }}{4a^{2}\kappa^{*}}[az]e^{-az}\\ -\frac{G^{*}B_{\alpha }}{4a^{2}\kappa^{*}}[a^{2}z^{2}]e^{-az}+C_{\alpha }e^{-az}, \end{array}$$ (60)

$${\tilde{T}}^{\beta }(z)=\frac{A_{\beta }}{2a^{2}}[az]e^{az}-\frac{B_{\beta }}{4a^{2}}[az]e^{az}+\frac{B_{\beta }}{4a^{2}}[a^{2}z^{2}]e^{az}+C_{\beta }e^{az}.$$ (61)

The corresponding horizontal velocities and pressures are

$$\begin{array}{l} {\tilde{u}}^{\alpha }=i(B_{\alpha }-A_{\alpha })e^{-az}-iB_{\alpha }[az]e^{-az},\\ {\tilde{u}}^{\beta }=i(B_{\beta }+A_{\beta })e^{az}+iB_{\beta }[az]e^{az}, \end{array}$$ (62)

and

$${\tilde{p}}^{\alpha }=2\rho^{*}\nu^{*}aB_{\alpha }e^{-az},\;{\tilde{p}}^{\beta }=2aB_{\beta }e^{az}.$$ (63)

The interfacial boundary conditions are

$${\tilde{\omega }}^{\alpha }=0,\;{\tilde{\omega }}^{\beta }=0,\;{\tilde{\mu }}^{\alpha }={\tilde{\mu }}^{\beta },$$ (64)

$${\tilde{T}}^{\alpha }={\tilde{T}}^{\beta },\;k^{*}{\tilde{T}}_{z}^{\alpha }-{\tilde{T}}_{z}^{\beta }=0,$$ (65)

$$(\mu^{*}{\tilde{\mu }}_{z}^{\alpha }-{\tilde{\mu }}_{z}^{\beta })+ia(\mu^{*}{\tilde{\omega }}^{\alpha }-{\tilde{\omega }}^{\beta })-ia\text{Ma}{\tilde{T}}^{\alpha }=0.$$ (66)

The interface boundary conditions determine the remaining six constants *A_α_*, *B_α_*, *C_α_*, *A_β_*, *B_β_*, and *C_β_*.

The velocity boundary conditions require *A_α_* = *A_β_* = 0, and *B_α_* = *B_β_*, and the solution to the thermal problem then gives

$$C_{\alpha }=C_{\beta }=-\left[\frac{\left(1-\kappa^{*}\right)}{4a^{2}\kappa^{*}(1+k^{*})}\right]B_{\alpha }.$$ (67)

The remaining boundary condition gives the dispersion relation,

$$\text{Ma}=8a^{2}(1+\mu^{*})\kappa^{*}\frac{\left(1+k^{*}\right)}{\left(1-\kappa^{*}\right)}.$$ (68)

For the values in Table I this gives Ma = 44.2 *a*^2^.
